# Face Time: Educating Face Transplant Candidates

**Published:** 2013-07-04

**Authors:** Brooke M. Lamparello, Ericka M. Bueno, Jesus Rodrigo Diaz-Siso, Geoffroy C. Sisk, Bohdan Pomahac

**Affiliations:** Division of Plastic Surgery, Brigham and Women's Hospital, Harvard Medical School, Boston, Mass

## Abstract

**Objective:** Face transplantation is the innovative application of microsurgery and immunology to restore appearance and function to those with severe facial disfigurements. Our group aims to establish a multidisciplinary education program that can facilitate informed consent and build a strong knowledge base in patients to enhance adherence to medication regimes, recovery, and quality of life. **Methods:** We analyzed handbooks from our institution's solid organ transplant programs to identify topics applicable to face transplant patients. The team identified unique features of face transplantation that warrant comprehensive patient education. **Results:** We created a 181-page handbook to provide subjects interested in pursuing transplantation with a written source of information on the process and team members and to address concerns they may have. While the handbook covers a wide range of topics, it is easy to understand and visually appealing. **Conclusions:** Face transplantation has many unique aspects that must be relayed to the patients pursuing this novel therapy. Since candidates lack third-party support groups and programs, the transplant team must provide an extensive educational component to enhance this complex process. **Practice Implications:** As face transplantation continues to develop, programs must create sound education programs that address patients’ needs and concerns to facilitate optimal care.

Face transplantation (FT) is becoming the treatment of choice for the most severe facial disfigurements. To optimize informed consent, compliance, and quality of life at every stage of FT, patients must be adequately educated on all aspects of this complex intervention. Ethical considerations have been raised on whether a patient's potential psychological vulnerability juxtaposed with the nonlifesaving nature of FT may lead to unintentional coercion and/or capacity for valid consent.^1^ A formal education program is paramount for FT candidates to fully comprehend the procedure's risks, benefits, and alternatives. Only when a patient understands this information can he or she provide true informed consent.^2^

Solid organ transplantation (SOT) experience has shown that, in addition to safeguarding the patient's rights, a multidisciplinary education program ensures patient satisfaction and continued support.^3^ Furthermore, maintaining relations with transplant team members and having a written resource to refer to when questions arise can help alleviate patient anxiety during the waiting period, which is often a time of uncertainty and worry.^3^^,^^4^ As for posttransplant concerns, a sound educational program is the most important, easiest, and cost-effective implementation to improve patient adherence to medication regimes.^5^ Nonadherence to medical treatment is the third leading cause of renal transplant rejection and contributes to increased incidence of acute rejection and potential graft loss in liver transplant recipients.^6^^-^8 Patient education and subsequent support from transplant teams also play an integral role in enhancing recovery and quality of life.^9^

Our FT program at Brigham and Women's Hospital (BWH) uses several approaches to education, with a goal of ensuring truly informed consent and encouraging proactive patient self-management and postoperative adherence to medications. These modalities include specialized written literature, education sessions with members of the transplant team, and multiple question-and-answer meetings for patients and their support system. Creating a deeper understanding within a patient's support system may facilitate adherence to medications, management of complications, identification of adverse events or symptoms, and psychological support.

Face transplantation is a pioneering application of advances in microsurgery and immunology, and it is recognized by its unique ability to restore near-normal appearance and function in patients with severe facial disfigurement. Because of the innovative nature of FT, there are no standard guidelines for many of the preoperative assessments, perioperative care, and postoperative follow-up components. Moreover, in contrast to well-established SOT programs, FT programs have few established protocols and data available to assess the educational needs of its candidates. Therefore, substantial effort has been dedicated to the development of an educational program that specifically addresses experience-based observations regarding the patients’ needs, concerns, and questions.

Here, we present our experience in developing an educational program for FT candidates and recipients at BWH, in particular, the development of a written handbook that will serve as a comprehensive source of information on all the steps and procedures that patients may encounter during the different stages of the transplant process. In addition, we present adaptations and provisions created specifically for the visually impaired, which represent a significant portion of the FT candidate population.

## METHODS

Previously, per suggestion of the institutional review board, candidates who presented for FT consultation at BWH received educational literature corresponding to kidney transplantation. No educational literature had been written specifically for the FT population; therefore, kidney transplant literature was deemed acceptably close as long as patients and their support network were advised on the differences between the two transplant types during consultations and interactions with FT team members.

As the volume of FT candidates increased, our group worked closely with the transplant administration team at BWH to generate an FT-specific patient handbook built upon the SOT patient education experience. Copies of kidney, heart, and lung transplant program handbooks were analyzed to identify applicable topics (Table 1). We then brainstormed with the multidisciplinary FT team to identify unique aspects of FT requiring patient education (Table 2).^10^ This multidisciplinary approach is one of the cornerstones of our FT program.^10^^-^12 In addition to consultations with transplant surgeons, plastic surgeons, and other specialists, our FT candidates and recipients frequently interact with other team members to address a number of issues, including, but not limited to, psychosocial aspects, insurance/medication coverage, rehabilitation, nutrition, how to inform other medical care providers of their transplant, media relations, and ways to adapt to life post-FT.

## RESULTS

Analysis of the SOT patient literature available at our institution allowed us to identify unifying themes, topics, and concepts that merited inclusion in the FT-patient handbook (Table 1). The FT handbook was organized as described in Table 3. We omitted information on medical conditions that may lead to an SOT, as well as the causes of severe facial disfigurement because these are often of traumatic nature and their discussion may lead to psychological distress. Instead, the FT handbook highlights the functions and abilities that patients could regain after the intervention, thus associating the handbook with pleasant and hopeful thoughts.

Patients encounter unique psychological challenges throughout the FT process. For this reason, they interact with psychiatrists and social workers as early as the evaluation stage. Information on stressors that patients and their support systems may encounter is included, especially on coping with public attention, new facial appearance, and transplant rejection. Identifying these issues in the handbook helps patients realize that these stressors are not unique to them as individuals and may contribute to early identification and intervention for stress or anxiety (Table 4).

Meetings with the hospital's media relations team help patients and their support networks handle media attention after FT. Unlike SOT, FT is a novel therapy, and the media maintains a high level of interest in any recipient of an FT.

**Figure 1:**
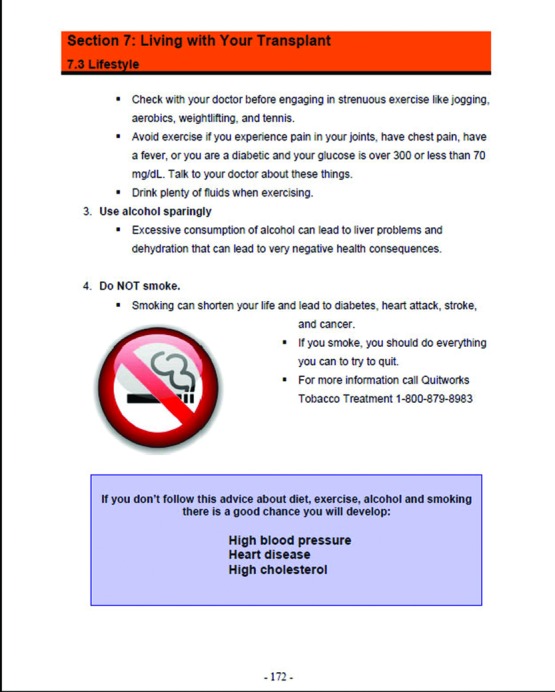
A sample from the 8.5 × 11-in handbook that showcases the use of large margins for annotations, graphics, color, and large font to make the handbook aesthetically pleasing.

To avoid overwhelming patients, the handbook must be easy to understand and concise. The language is simple, and medical jargon is avoided whenever possible. Certain terms and procedures that patients need to familiarize themselves with are included with brief explanations. While the handbook is large (181 pages) and covers a broad range of topics (Table 3), every effort was made to render it aesthetically pleasing and appealing by using large font sizes, graphics, color, and wide margins (Fig 1). There are also spaces and blank pages for the patient to write notes, calendar templates to track visits, and forms to log postoperative health status.

Throughout the text, the handbook reiterates the theme of teamwork among the patient, his or her support system, and the transplant team to ensure the most positive outcome possible. The introductory welcome letter to the patient states:

As outlined in this manual, successful facial transplantation requires a coordinated, multidisciplinary team effort. You—the patient—are also an important member of a successful facial transplant team. Becoming educated prior to the transplant and actively participating in rehabilitation enhances the chances for a good outcome after transplantation.

The theme is once again reiterated when addressing barriers to compliance:

Please speak to your transplant caregivers if you believe you are facing barriers to compliance—whether these barriers are within or beyond your control. The transplant team, including the social worker and psychiatrist, will work with you and your family to help you overcome these barriers.

It maintains an encouraging, nonjudgmental, and positive tone—even when addressing important issues like medication adherence. Even so, the handbook remains realistic regarding the serious aspects of the FT process and emphasizes risks and what may happen if the graft is rejected. The book also encourages patients and their support system to maintain an open dialogue and ask questions to the always-available transplant team members.

## DISCUSSION

Face transplantation is a relatively new procedure; hence, there is no published work to date advising centers on adequate education for patients. Creating a program that patients and their support system will not only participate in but also benefit from is important to optimize informed consent, pre- and posttransplant quality of life, and adherence to postoperative medications. We have presented a description of the efforts to ensure that our center's FT candidates receive the educational resources required to achieve these objectives.

Waterman et al^13^ surveyed a cohort of kidney transplant recipients and found that the main sources of health education were discussion with medical staff and written literature and that the most frequently requested topics of transplant education were discussion of the evaluation process, common concerns of transplant recipients, and a schedule of medical tests required during the transplantation process. It is our belief that the FT patient population would share these concerns; therefore, inclusion of these topics may prove useful to our patient population and their support system.

Compliance with follow-up care and immunosuppressive medications is fundamental to transplant survival.^14^^-^16 A meta-analysis estimating the impact of nonadherence to immunosuppressant medications in renal transplant recipients resulted in a 7-fold increase in the odds of graft failure.^16^ Within the face and hand transplant recipient population, cases of nonadherence to immunosuppressant medications have been reported, resulting in graft loss and even death.^17^^,^^18^ These cases served to highlight the importance of pretransplant screening and patient selection. It is our strong belief that these examples should also stress the significance of patient education to prevent noncompliance, identify barriers, manage and promote realistic expectations of the procedure and its results, and strategize ways to optimize adherence. Compared with compliant recipients, SOT recipients who are noncompliant often harbor higher distress, misconceptions and poorer understanding of the transplant, greater concerns about medication adverse effects, and lower beliefs regarding the benefits of transplant.^19^ Furthermore, it has been suggested that patients form complex ideas about their transplant and medication regime and try to manage concerns about medication side effects and transplant-related fears such as graft failure by modifying their adherence.^19^ A program that emphasizes teamwork and open communication with transplant care providers can circumvent the formation of these complex beliefs and promote adherence to medications.

Face transplantation involves a long, exhaustive evaluation process followed by a waiting period of uncertain duration and, quite possibly, high anxiety. Thus, informed consent in our patient population is a continuous process of communication rather than a single event. At BWH, patients provide informed consent before screening. However, it is openly communicated to patients orally, in the handbook, and in the consent form itself that they may withdraw consent any time before FT. An important factor relayed to patients is that there is no safe way to withdraw consent after the transplant procedure. By using an education program that begins in the screening/evaluation stage and continues after the surgery, we can avoid unintentional coercion of psychologically vulnerable patients. Furthermore, during the evaluation stage, patients meet with a study psychiatrist; they then obtain a second opinion from a study-independent psychiatrist who serves as patient advocate to ensure capacity for consent. The patient handbook and one-on-one sessions with transplant staff also reiterate the alternative therapies available to candidates.

Many SOT recipients turn to public organizations, support groups, and Internet sites for information and encouragement.^3^ Fewer than 25 FTs have been performed to date; these resources do not currently exist for FT patients. Consequently, the education program must address all potential questions and concerns the patients and their support systems may encounter and must not be limited to only the medically related issues—we must be our patient's support community. Education programs and materials must address as many common issues as possible. Moreover, team members must establish a rapport with patients so that they will comfortably ask questions that may or may not have been answered in the handbook or in previous one-on-one education/discussion sessions with FT providers.

One unique aspect of FT is the extensive evaluations necessary to identify suitable candidates. Although it is verbally expressed, patients often underestimate the number of appointments, tests, and steps in evaluation. By including an outline in the handbook, teams can provide patients with a written resource to refer to while at home; this outline can also serve as a checklist of sorts, allowing them to precisely track the steps in their screening process.

Also included are common psychological stressors encountered by FT candidates and recipients. Face transplantation recipients are often predisposed to psychological sequelae more so than SOT recipients because of the aesthetic nature of their transplant and the external location of the allograft. The face is often seen as the most important physical attribute that forms one's identity.^20^ Drastic changes to a person's face, such as disfigurement or FT, may lead to a perceived altered identity and can cause stress.^12^ Care must be taken to manage patients’ expectations and avoid disappointment with the appearance or function of their allograft. In addition, the general public maintains interest in documenting the lives of FT recipients, and so the educational program includes discussions with media relations specialists to prepare patients and their families for dealings with the media.

Face transplantation has a very unique advantage over SOT, when monitoring for immune rejection. Solid organ transplantation involves internal vital organs, and episodes of rejection can only be monitored through invasive medical procedures after manifestation of symptoms that may not be easily identified as rejection by the patient. Conversely, FT rejection results in easily discernable redness and swelling. To address the signs and symptoms of rejection, the handbook includes sensible photographs of a prior FT recipient experiencing an episode of acute rejection. Early identification of acute rejection and the subsequent early administration of medications may lead to increased chances of therapeutic success.

In addition to the easily anticipated aspects of FT, the handbook provides suggestions for optimizing quality of life after FT. Patients often overlook the less intuitive aspects of transplantation, such as psychological and social implications. Lung transplant recipients have expressed desire for more information in this area.^21^ Inclusion of these topics in the handbook helps prepare patients for life after FT. We included information on diet and exercise to maintain a healthy weight, safe international travel, and other situations to which FT patients must give special consideration. Whereas SOT programs place much of their focus on avoiding weight gain following a transplant, many FT recipients are underweight before transplant, as their disfigurement may complicate nutrition. Restoration of oral competence after transplant often results in some weight gain. Our handbook focuses on healthy weight gain and maintenance to decrease risk of other medical problems, like hyperlipidemia or diabetes mellitus.

Educational materials that lead to optimal learning are written in a fifth- to eighth-grade reading level.^22^^-^24 To avoid overwhelming patients and to maximize learning, the handbook was composed using very simple and understandable language. This is particularly important when discussing potential medical problems or procedures that may be associated with immunosuppression.

Diabetes is a condition in which the pancreas cannot produce enough insulin (a hormone) to control the metabolism of glucose (sugar) in the body. People with diabetes tend to have high blood glucose levels. It is very important to control glucose levels and avoid high levels to prevent damage to the eyes, heart, and kidneys.

The handbook contains a table of contents, an index, chapter summaries, and color-coded chapters to break it down into chronological sections that patients can read on their own time. This allows the handbook to be used as a resource or reference guide, rather than a required reading. The handbook is quite comprehensive, and the inclusion of graphics and images to break up the text make it visually appealing and less intimidating.

The mood in which the information is delivered is also important in optimizing the learning experience. If patients perceive a lack of interest from their health care providers, there is an increased risk of nonadherence.^5^ It is essential that FT team members remain optimistic and encouraging when communicating with patients and their support systems. This tone reverberates in the handbook. Creating a supportive learning atmosphere increases confidence, patience, and respect and promotes an active learning role for the patient.^25^ Renal transplant patients have also expressed desire for continuity with team members providing education. Several recipients felt that becoming familiar with the educator facilitated learning by building relationships and not having to relate to a new person.^25^ To help FT patients build relationships and familiarize themselves with members of the team, the handbook includes a “Who's Who” section with names, contact information—if necessary—and descriptions of each member and their role in the patient's care; team members are identified by first name whenever appropriate. Besides fostering the team mentality, this provides the patients with a roster of team members and helps them identify the best person to address a specific question or concern.

Like many aspects of the FT protocol, our education program has adapted to individual patient needs. As 2 of our FT recipients are visually impaired,^26^ their educational program relied heavily on one-on-one verbal teaching sessions and their support system reading the handbook to them. Efforts to convert our written handbook into an audio recording are under way; this resource may allow visually impaired patients and future candidates to listen to the educational information at their own pace and convenience. Further multimedia resources and educational formats have been considered. Kidney transplant candidates have suggested online videos for patients and their support system, open group discussion sessions, and an agenda/formal letter allowing patients and their families to know what to expect.^27^

## CONCLUSION

The BWH Face Transplantation Program has observed a need to provide a comprehensive patient educational experience. By developing written materials, encouraging open discussions with a multidisciplinary team of specialists, and identifying individual difficulties and concerns, every effort is made to inform patients and their support systems of every aspect of FT. As experience grows, quantitative studies to evaluate responses to educational programs may provide significant results, which can lead to an optimized and improved patient experience.

## PRACTICE IMPLICATIONS

The BWH Face Transplant Program has developed a multidisciplinary approach to educate patients and their support systems. Since external FT resources or support groups are not yet available, a written handbook provides the patients with a comprehensive resource that may always be at their disposal. This may give patients the tools and knowledge necessary to maximize their inpatient experience, recovery, and overall health and quality of life.

## Figures and Tables

**Table 1:** Analysis of the BWH solid organ transplant handbooks identified several universal topics to any kind of transplantation*

Universal themes, topics, and concepts in the BWH solid organ transplant handbooks
Guidance to the evaluation process
Tests and laboratory results
Information regarding the organ bank
What to expect at the time of the transplant
Information on the procedure
Postoperative instructions
Explanations of posttransplant medications
Risks in transplantation
Optimizing quality of life after the transplant

*BWH indicates Brigham and Women's Hospital.

**Table 2:** Some topics not addressed by solid organ transplant educational materials, but included in the face transplant handbook

Psychiatrists	Psychiatric evaluation and follow-up facilitate psychological well-being. Psychiatrists and social workers also help in identifying and developing coping strategies for any stress or anxiety.
Media relations	Information is provided to the patients and their support systems in handling any subsequent media attention they may receive after their transplant.
Rejection of the graft	Sensibly chosen and nongraphic pictures and descriptions of symptoms of acute rejection are provided to help the patient understand and identify when they may have an episode of acute rejection.
Information on transplant biopsies	Frequent skin biopsies are required to check for histological signs of rejection. Information is provided on the procedure, how to prepare for, and any complications of having a biopsy.
Benefits of face transplant	Pictures and descriptions of the facial functions that may be gained with a face transplant.

**Table 3:** Components of the face transplant handbook at BWH*

Components of the face transplant handbook	Description
Who's Who—list of the BWH face transplant team	Team member's role and contact information. Includes both health care providers and logistic coordinators, such as the finance and patient coordinators.
Social work and face transplant	Covers topics of discussion between social worker and patient, that is, compliance, coordinating benefits, health care proxies, returning to work, Family Medical Leave Act, and medication coverage.
Tissue typing and matching	Explains how donor-recipient matches are made.
Deciding and planning for transplant	Advises on making the decision on whether or not to proceed with an FT and how to plan for the time when a donor is available.
Procedures at the time of transplant	Details what to do when a donor is available, signing consent forms, and the surgical procedure.
Rehospitalization instructions	Provides guidance should patients need to be readmitted for complications.
Writing to the donor family	Provides information for patients who wish to write to the donor family.
Posttransplant visit schedule	Provides a detailed list of the different health care providers patients will have to meet postoperatively and how often they can expect to see them.
Instructions for coming to the clinic	Provides instructions on medications, laboratory work, prescription refills, and getting to the clinic.
Medical information for transplant recipients	Provides information on rejection, infection, biopsies, medical problems associated with transplants, future participation in clinical research, and relevant information to provide to other doctors not familiar with FT.
Information regarding over-the-counter medications and drugs that interact with immunosuppressive medications	Provides information on contraindicated over-the-counter medications and supplements. Lists the drugs that interact with immunosuppressive medications.
Medication schedule	Provides a sample schedule to help the patient visualize and plan how and when they should take their medications.
Living with a face transplant	Gives tips on maintaining healthy weight, eating healthy, appropriate exercise, information regarding sexual activity, and pregnancy posttransplant
Travel tips	Provides information on medical logistics when planning to travel: ensuring insurance coverage, notifying the transplant team, locating a transplant center near the destination, list of vaccines for transplant patients.

*BWH indicates Brigham and Women's Hospital; FT, face transplantation.

**Table 4:** Unique psychological challenges of face transplant candidates and recipients

Negative feelings, being upset, or unsettled about having a new face or changed face, since the face is a large part of one's identity.
Anxiety or fear of side effects and complications of the surgery, such as an uneven face—where the two sides do not exactly match, limited facial movements, drooling, trouble eating, chewing, or swallowing.
Feelings that friends and family members are disturbed by or uncomfortable by the changed appearance. If people, especially loved ones, have a hard time accepting a patient's new appearance, it may be distressing.
Concern regarding rejection of the transplant, the possibility of having a damaged face again, or an even worse appearance, if the transplant fails.
Stress, anxiety, or upset over the loss of privacy that can result when news media becomes involved.
Anxiety or worry if the patient is found ineligible for transplantation.
Disappointment if the patient is ineligible after screening.
